# Collaborative agency to support integrated care for children, young people and families: an action research study

**DOI:** 10.5334/ijic.1171

**Published:** 2014-05-08

**Authors:** Kaz Stuart

**Affiliations:** Evaluation at Brathay Trust; University of Cumbria, UK

**Keywords:** integrated, collaborative, agency

## Abstract

**Introduction:**

Collaboration was legislated in the delivery of integrated care in the early 2000s in the UK. This research explored how the reality of practice met the rhetoric of collaboration.

**Theory:**

The paper is situated against a theoretical framework of structure, agency, identity and empowerment. Collectively and contextually these concepts inform the proposed model of ‘collaborative agency’ to sustain integrated care. The paper brings sociological theory on structure and agency to the dilemma of collaboration.

**Methods:**

Participative action research was carried out in collaborative teams that aspired to achieve integrated care for children, young people and families between 2009 and 2013. It was a part time, PhD study in collaborative practice.

**Results:**

The research established that people needed to be able to be jointly aware of their context, to make joint decisions, and jointly act in order to deliver integrated services, and proposes a model of collaborative agency derived from practitioner’s experiences and integrated action research and literature on agency. The model reflects the effects of a range of structures in shaping professional identity, empowerment, and agency in a dynamic. The author proposes that the collaborative agency model will support integrated care, although this is, as yet, an untested hypothesis.

## Introduction

In the past decade there has been a host of policies that have increased the pressure on practitioners to collaborate in the children's workforce. The ‘children's workforce’ is a broad term that encompasses many different and diverse professions in health, education, social care, child care, policing and justice, leisure and culture. The form of working that resulted from these professions coming together was called ‘integration’ [1] and requires people from across the children's workforce to share information, work together, use common tools and sometimes co-locate, overcoming professional differences and boundaries. The success of these policies and strategies in terms of integrated care for end users, and cost efficiencies is debated [2, 3]. Yet the rhetoric was formidable, with 47 policies, guidance and bills referring to integration in the 1990s and 88 in the decade from 2000. The message was clear:

integration is the glue that bonds the entities together, thus enabling them to achieve common goals and optimal results. [4]

The events leading to integration are well documented. Eight factors drove integration in the UK from the 1940s onwards. Soon after the establishment of the Welfare State came the death of 12-year-old Dennis O'Neill due to the abuse and neglect of foster carers. This led to the 1948 Children Act and development of the first local authority departments responsible for children. The death of Maria Colwell at her parent's hands in 1973, despite 50 official visits to her home, led to the establishment of the modern child protection system. In 1988 Jasmine Beckford died, abused and malnourished despite 66 carers involvement in the case. The 1989 Children Act heralded the start of a new era, with the welfare of children a statutory priority, along with the right for children to have their voices heard. This was in accordance with the United Nations Convention on the Rights of the Child [5] that was also signed and made legal 1989. Despite such legislation, Victoria Climbié was tortured and murdered in 2000. The Laming Inquiry [6] made 108 recommendations that led to the Every Child Matters (ECM) Green Paper [7]. All these abuse cases had a lack of interagency communication and information sharing highlighted as prime failings. As such, the ECM paper focussed on bringing about integrated working. It promoted integrated working through the unification of services under the Children's Workforce Development Council [8] and a Common Core of Skills and Knowledge. A Common Assessment Framework was launched along with an information-sharing database called Contactpoint, shared tools for all professionals to use. Lead professionals were appointed to ensure that services were configured around the needs of individual children and Directors of Children's Services presided over multiagency Children's Trusts, ensuring the integration of services at a local authority level. The drive for integrated outcomes and services embodied in ECM became policy in the Children Act 2004 [9] and 2007 Children's Plan [10]. The stage had been set for integration on a scale never seen before. Kellett [11] surmises in her book that the agenda has brought benefits to England, increasing understanding of the need; to communicate, to plan holistically around the needs of the child, to intervene earlier in the lives of children and to listen to children.

Post the 2010 General Election, the Coalition Government revoked the need for statutory integration and the language of the previous government was banned [12]. Just as ‘integration’ seemed to be waning, year-on-year economic spending reviews drove a second wave of ‘partnerships’ and ‘collaboration’ resulting from the pressures of reduced budgets for services, rather than statutory requirements. Integration, collaboration and partnership were presented as the way ahead in a climate of economic paucity [13].

England was not alone in its drive for collaboration. The four home countries of the UK all adopted collaboration and integration. In Scotland the initiative was called ‘Getting it Right for Every Child’ [14], whilst Ireland's Ten Year Strategy [15] for children and young people contains a similar recognition for the need to integrate services around the needs of children. Wales also has a requirement for collaboration and partnership working to drive outcomes for children. The UK's fervour for integration was perhaps not paralleled overseas. The Centre for British Teachers (CfBT) review of international integration found that:

Although a majority of countries and sub-national jurisdictions (34 of the 54 in the sample) have shown some level of commitment in policy terms to a joined-up or collaborative approach, very few have emphasised the centrality of integration along UK lines [16]. Alberta, Malta and the Netherlands were the notable exceptions as they did have integrated services like those found in the UK. All of the countries in the CfBT review were, however, found to be on a journey towards joined-up, collaborative or integrated services. [17]

Against this context, this study sought to understand the lived experiences of practitioners trying to deliver integrated care. There were multiple changes, restructures, guidance and tools, but the extent to which these helped practitioners to collaborate to provide integrated care was unknown. This research sought to understand what such collaborative practice looked like, and the extent to which it helped deliver integrated care. The professionals involved in all the cases above probably understood how to communicate with other agencies and are likely to have understood the necessity for integrated working, so why was it so evasive? The notion of ‘agency’ emerged from the research and was the central concept that explained what was happening. Agency refers to the awareness, choices and actions of an individual striving for what they need in the world. If extended to a group, rather than to an individual, the concept had potential to explain that the multiagency groups trying to achieve integration could be hindered by a lack of collective awareness, collective decision making or collaborative action. Whilst the concept emerged late in the research process, it is introduced here to frame the research. Professionals in the children's workforce with collaborative agency would be active subjects, able to make things happen, rather than passive objects to whom events happened [18]. Action does not necessarily mean activity – choosing to do nothing is an action [19]. What is important is that the collaborative team would assess the context, make choices, and use their power and capacities to interact with the world around them [19].

## Methods

The study commenced with a simple question about how people collaborated in the children's workforce, an interest that extended from the author's academic work teaching a Masters course in integrated service leadership. The four action research cycles iteratively developed and refined the research and led to the examination of agency in a collaborative context. The research was initially funded by the University of Cumbria, but employment changes for the author meant that the study was eventually self-funded. Changes in employment were not unique to the author and also occurred for many of the research participants, adding discontinuity and complexity to the study. Despite this, the research does represent multiple perspectives on collaboration within a three-year timespan for the children's workforce in one local authority.

The study used action research as it was investigating a lived experience, the social practice of collaboration [20]. A second key defining feature of this research, and action research, was its participatory and democratic nature, working with practitioners to make meaning from their experiences, rather than drawing data ‘from’ them. From this perspective, the research sought to improve the professional lives of people trying to deliver integrated care, and ultimately, to improve the lives of their beneficiaries [20]. Another dimension to the participatory approach of action research is the shift of power, placing practitioners as the experts on their experiences, rather than privileging the role of the academic in theorising what they observe. A third feature of this study, as action research, was its efforts to achieve, praxis, continually bringing theory and practice together to further practice [21]. The final feature of action research was the methodological flexibility that it afforded the study, and that was necessary to construct, and deconstruct collaboration in a range of settings with a range of participants. The four action research cycles included: autoethnography [22, 23]; developmental research workshops [24]; creative tools [25–28] interviews and observations [29]. These tools became a methodological bricolage [30], a collation of a range of methodological tools.

Validity and reliability are indeed quantitative terms, and so applying them in qualitative research is problematic. Instead, this research was credible, confirmable, crystalised and unbiased [31]. Participant validation was used to ensure representativeness and credibility, that is, that the data sets represented the experience of the participants. Confirmability, that is, that the data corpus reflected the sum of the range of experiences of collaboration was achieved by the depth and duration of the study, spanning three years and involving 127 participants. The iterative research cycles, analysed consistently in an inductive approach, all confirmed that the emerging conceptual framework reflected experience. Periodic participant checks also helped to confirm that the interpretation reflected their experience. The combination of multiple practitioners’ perspectives, in different settings, with different data collection tools ensured that the different aspects of collaboration were captured and reflected in a process of ‘crystalisation’ [32]. Bias was avoided with an open, inductive coding process and there was a clear audit trail in Atlas-Ti (qualitative data analysis software). Codes were checked and re-checked in a constant comparison process [33]. Scrutiny of the analysis of the data by peers and supervisors also helped to ensure that the process of analysis was robust and unbiased.

The first action research cycle was practitioner action research investigating the author's practice as a leader of a collaborative team of 20 people [34]. This was first person action research [35] as the author examined her own practice in an autoethnographic account. The second action research cycle involved the analysis of safeguarding with five locality management teams across a local authority involving 66 multiprofessional managers (a 66% sample including health, education, family services, careers, social care, further education, children's services and policing). Activity system analysis was used to identify the complexity contradictions of the system [36]. The third action research cycle was conducted across a series of participative workshops with 12 members of the team in cycle one that had volunteered to participate following the findings that the author presented to them at the end of cycle one (a 50% sample health, education, family services, social care, further education, children's services and policing). This research cycle was dialogical, involving democratic discussion and it employed creative elicitation techniques, such as physical team mapping, and activity systems mapping. This combination of tools employed over a length of time created an in-depth understanding of this team's experiences. The fourth and final action research cycle involved interviews with 11 individuals from across the children's workforce (including health, education, youth work, social care and children's services), and observation of a large task and finish team (18 people including health, education, social care and children's services) who were trying to reduce bureaucracy in referrals in children's services (a 0.06% sample of the workforce). This action research cycle allowed the author to pursue the lines of enquiry that emerged in earlier cycles.

Braun and Clarke's [37] analytical method was applied to the data using thematic analysis within the computer aided qualitative data analysis software. After the initial coding, analysis and action within each action research cycle, the data corpus was reviewed and found to contain four key themes in the data corpus that were not all visible in the analysis of the individual action research cycles [38]. These were the importance of contextual awareness, the significance of individual and group identity, the necessity for teams to be empowered and ‘agency’.

Ethical issues were carefully considered. Confidentiality and anonymity were of particular importance given the high profile nature of protecting children's well-being. For this reason, the local authority characteristics, job titles and names are not used to protect participants. The extent of participation was constantly renegotiated, as were the research activities, and informed consent and participant validation became on on-going process. Ethics could not be covered at the initiation of each action research cycle but were continually present.

There were methodological issues to overcome throughout the study. It was not always possible to engage participants in a participatory research process due to constraints of time that they or their employers imposed. As a result, the level of participation varied greatly across the action research cycles. Second, the practitioners did not always view themselves as experts and did not wish to be positioned as such. They engaged with the research to be provided with answers, not to develop solutions themselves. This took some careful negotiation. The professional roles of the participants, and of the author also changed throughout the research period. People, and whole teams, were made redundant due to spending cuts, and this often changed the relationship between the researcher and participants from researcher, to leader, to peer. Researcher relationships were a constant dynamic and cause of tension across the study. The range of creative tools, changing relationships and four action research cycles therefore led to ‘messy’ research [39], research that is not linear, sequential or simple. The final methodological limitation is the highly contextualised nature of this study, located as it is in one local authority in the UK. It is hoped that enough is presented in this paper to allow the reader to judge how generalisable the resulting model of collaborative is.

It is impossible to present the entire data corpus in this paper, instead selected literature and illustrative data are presented that demonstrates how the five key themes came together in the model of collaborative agency, a model that could support integrated care. The action research cycle that each data excerpt came from is indicated in brackets following the quote. This paper hopes to add to the on-going debate as to the fundamental principles of integrated care [40].

## Results and discussion

### The importance of contextual awareness

Throughout the action research cycles, practitioners had talked about the ‘barriers’ that they encountered to collaboration. Occasionally they also talked about the things that enabled them to collaborate. What they were describing were the contextual structures that they operated in. The data showed that these existed at a number of interconnected layers, the macro, meso, micro and beneficiary levels. Awareness of these enabling and constraining interacting layers of structure was vital for practitioners to navigate integrated care.

Micro barriers included all the things that happened in day-to-day practice, such as the pressure of time versus workload: ‘Everyone is now overworked as they have less admin support and so much more in each job description’ (ARC3). Colleagues were equally described as enabling or constraining daily practice: ‘Helpful managers allow autonomy and mainly let me get on with my job. Unhelpful school managers want information useful to them, but that is useless for the children, within the day, which causes organisational difficulties for me’ (ARC4). There was a host of issues at this level: ‘A lack of understanding of each other's ‘language’ and/or belief systems, protocols, etc. can cause difficulties, also poor understanding of how each other's departments work, i.e. what hoops we each have to jump through, and why things can't happen yesterday’ (ARC2). Generally however, the practitioners (whether front line or senior) felt that they had some control over contextual factors at this level. These were interactions with other people, networks, relationships, ‘know how’, ‘know who’, and ‘know what’, daily taken for granted and newly negotiated forms of practice. The micro level included ways in which practitioners saw themselves; others; and the structures that they were situated in, and is influenced by the ways in which they are framed, viewed and acted on by all the other members of the system.

The meso level is the organisational level of the system. It included organisational culture, policy and structures. These were overt and covert. It also included the professional backgrounds and disciplines of practitioners, their frames of practice, practice guides and other espoused forms of practice. Sometimes meso structures were imposed by people's own organisations, or by the organisations they were providing integrated care with: ‘The school said that they couldn't do anything with one child because they were snowed under’ (ARC3), and: ‘We keep bureaucracy to a minimum so that we can deliver effectively to young people’ (ARC3). Despite the level of these structures, there were still some practitioners who felt that they had the scope to exploit enablers, and overcome barriers: ‘Sometimes we did things in spite of the Trust Board, the managers always cited them as a barrier, and I would get them to almost forget about the Children's Trust Board and just get on with the job’ (ARC2). The meso level included ways in which organisations and professions saw themselves; others; and the structures that they were situated in, and it is influenced by the ways in which they were framed, viewed and acted on by all the other members of the system.

The macro level includes the explicit national frameworks for practice such as the Acts of Parliament, policy documents and guidance. It also includes the tacit discourses and hegemony that shape and influence practice. Practitioners felt that they had some, albeit less, control over these contextual structures: ‘I'm selective and do what I want to do. I need to. It sounds terrible. Ofsted for example, many have to change practice to meet the requirements to pass, but I think that it's just minimum requirements, we should be better than that, so I meet and go above that because we have to and should. On Government Acts - I don't give my opinion, I just implement them with good grace because we have to’ (ARC4). Awareness of these contextual factors varied, but there was a general sense that it was significant as: ‘Professionals are really affected by all the changes and political stuff’ (ARC2). The macro level included ways in which politicians and ‘society’ as a whole saw; themselves, others, and the structures that they were situated in. It was influenced by the ways in which they were framed, viewed and acted on by all the other members of the system.

The practitioners also felt that there needed to be ‘join up’ between the mandates at a national level, organisational policies and front line work. This was, however, experienced as fragmented, creating possible angst as the following data shows: ‘The real work is to connect leaders with the ground, their strategy needs to be rooted in the reality of the front line, or nothing will ever work’ (ARC2).

Another level of structure was also apparent – beneficiaries. The people that practitioners were working for, the beneficiaries of care were positioned, and positioned themselves as open to change, dependent on or resistant to help. These beneficiary structures also involved the ways in which the beneficiaries saw; themselves, others, and the structures that they were situated in. It was influenced by the ways in which they were framed, viewed and acted on by all the other members of the system. The importance of positionality was highlighted by Le Grand's work on families in social welfare as ‘pawns’ in a chess game [41]. The research participants were very aware that they could not enable beneficiaries to move on unless they were open to change as demonstrate in this data excerpt: ‘I can't make them, they have to want to, it can be so frustrating!’ (ARC3). The extent to which the beneficiaries felt empowered would have an effect on micro level practice, as all practitioners strove to empower beneficiaries to help themselves in a sustainable and sustaining way.

The model of collaborative agency needed to account for the interaction of these four levels of entwined structure and people's actions. Beneficiaries, micro, meso, and macro level structures all enable and constrain one another in mutually reinforcing and conflicting ways. The four structures were equally important, and they were not in a linear or hierarchical structure themselves. The celtic knot seemed a useful symbol for the interaction of these four areas, with the ‘thread’ of people's actions and the influence of contextual structures flowing from and through each area in ways that shaped and were shaped by one another. The knot has four areas representing each of the four layers of structure, they are intertwined showing that they are mutually influencing, rather than hierarchical.

**Figure 1.**
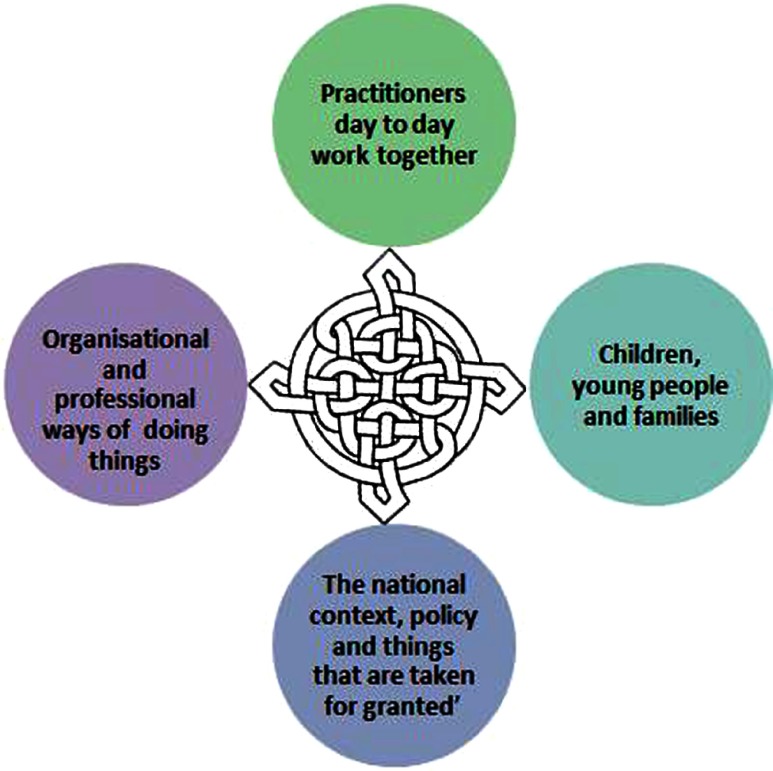
‘Structure at Multiple Levels’ in the collaborative agency for integrated care model.

Awareness of this structural context was key to practitioners operating successfully within it. The more awareness they had, the more scope they seemed to have for action. I will return to this theme within the discussion of agency (Figure 1).

### The significance of identity

Working together to achieve integrated care seemed to involve the negotiation of multiple identities. There are always personal and professional identities invoked in any work setting, and in this collaborative setting the identities were additionally multiprofessional and collective: ‘When you ask for my views, do you mean as a single professional, as a member of my organisation, or as a member of this multiagency team?’ (ARC3). These identities incorporated the professionals sense of self, professional self, self in a group (alongside many other day-to-day social selves), and self in a multiprofessional group. These identities did not seem to be fixed or constant, but were continually changed and revised, linking to the theoretical work of Hornby and Atkins on identity in multiprofessional teams [42]. Practitioners configured who they were in relation to the other team members, and the nature of the team, and hierarchies clearly existed: ‘As an educational psychologist I am used to getting my voice heard, but know that is not right in all contexts’ (ARC4). There is much literature documenting the difficulties of identities in multiprofessional teams including pragmatic differences such as pay and psychological differences such as professional status [42–44]. These difficulties were evident in this research. For example, participants commented that: ‘they are still in professional silos’ (ARC1). Creative exercises elicited tacit stereotypes that contradicted the values of collaboration expressed explicitly by some of the teams who participated. Health professionals in the Children's Trust were described as: ‘the biggest object but it's small and empty’, and: ‘it's a dark and unfathomable shape that is incomprehensible’ (ARC2). The collective multiprofessional identity was in part governed by the structural context. To use an extreme example, a team that was given no authority, and was comprised of people whose organisations saw no value in joint work had a limited collective multiprofessional identity (ARC2). For some practitioners, the other priorities in the rest of their mono-professional lives prevented them from fully participating in the collaborative work of integrated care: ‘Maybe this is why there are challenges, as I have so many other areas to deliver in, so it's never central. so not everyone in the team can do as much or contribute equally as it's just not their complete role’ (ARC2). All the participants knew that they needed a shared multiprofessional identity, but these difficulties seemed to exist nonetheless. Nor did they have to be shared, the views of one member of the team about the dress code of colleagues would create disharmony (ARC3), and as such, identity was framed by a combination of participant's views about themselves, others and the group, and by the views of the other team members about the others and the team.

When teams felt aligned they clearly found it easier to work together: ‘This feels collaborative. It is certainly easier to work collaboratively with the people that I get on with best, and that means having shared values and beliefs and working styles, and knowledge at the same level’ (ARC2). Conversely, when commonality was lacking, distrust could creep in, and less was achieved: ‘There is a real lack of trust now, I think that trust is the key to collaboration, and now it's gone there is less and less collaboration’ (ARC3).

The model of collaborative agency needed to reflect a collective account of multiprofessional identity that was shaped contextually as identity was evidently a factor in identity development (ARC4), and that in turn affected empowerment and agency, as having a positive identity influenced people's actions and vice versa (ARC3 and ARC4). As such, identity became the second item in the model.

**Figure 2.**
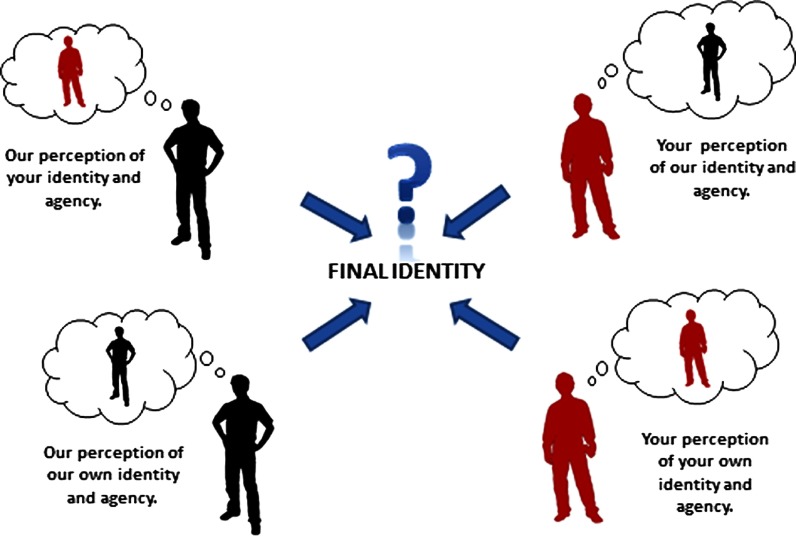
‘Collective Professional Identity’ in the collaborative agency for integrated care model.

Just as the people in the structures could be positioned in certain ways by policy, practitioners and beneficiaries, so identity is shaped by perceptions. The view that teams had of their own identity influenced it, as did the views that other practitioners and teams had of it. The perception that the participant's teams had of other practitioners and their teams influenced identity. The collaborative identity was therefore individually and collectively constructed as viewing others as ‘more able’ or ‘more powerful’, led to a reflexive positioning as ‘unable’ or ‘weak’ (ARC4). This is a crude characterisation of a sophisticated process of positioning. As these components were always shifting, so the collective multiprofessional identity was also fluid. The four ‘perceptions’ of identity are shown in the four corners of the diagram of collective multiprofessional identity. The resulting identity is shown as the central area in Figure 2, contingent on the interplay between the four perceptions.

‘Collective multiprofessional identity’ was unstable, in a dynamic with the other component parts of this model. The nature of the participant's team's identity affected the extent to which they felt empowered to act, and so empowerment is the next part of the model.

### The necessity of empowerment

Empowerment was significant in each action research cycle. There were multiple references to other people's levels of empowerment: ‘We are disempowered by the Children's Trust Board not allowing us responsibility and by the lack of power that individuals have in their organisations’ (ARC3). Participants connected empowerment to action, they saw it as enabling them to achieve integrated care: ‘I didn't think that people were empowered as they are not able to make changes’ (ARC4), and: ‘It also depends on leadership and empowerment - some leaders won't let you get on with it, or won't empower you to take action, so people might want to do stuff but can't’ (ARC4). Empowerment stems from Freire's work alleviating oppression [45] it literally means having power [38]. Some people needed to have power granted to them in order to achieve tasks (ARC1), others claimed power whether they were granted it or not (ARC2), and yet others ignored the power that they had been granted (ARC4). It was a complex process as demonstrated by this data excerpt: ‘Well there is power in terms of having the validity to act, and they certainly had that, but then they also needed to accept that power and acknowledge that they can use it’ (ARC4).

The collective multiprofessional identity that participant's teams had incorporated their sense of their own power. If their actions were effective and they could see impact of their work in any of the structures (e.g. achieved outcomes for families, negotiated a new practice, developed the organisational culture, got a policy rolled out nationally), they had a positive collective multiprofessional identity and felt empowered (ARC4). The opposite is also true, and there was stasis in some of the teams, actions not leading to change, creating a disempowered identity as a result (ARC4). Empowerment was therefore influenced by the structures, identity and the outcome of previous actions.

Maynard's [46] empowerment framework was integrated into the model as it correlated with the data collected. The model shows individuals in reactive or proactive states. When reactive, the individual responds to external stimuli, and the person experiences being ‘done to’. When proactive, individuals are resourceful, feeling in control and able to ‘do’. This resonated very clearly with the positions of the research participants throughout the action research cycles.

The initial stage of empowerment is ‘sparking’ where practitioners sense that things can be different, they do not always have to act in the same way, they can make choices. The spark may be positive or negative, that is, I want this, or I do not want this. These positive and negative drivers for action were evident in the participant's teams. In Maynard's framework, this leads individuals to realise that there is potential and possibility, creating a ‘wanting’, a real desire to achieve that new possibility. The empowered individual then commits to the change, and may need skills to enable them to achieve what they want. This resonated with the team effecting a reduction in bureaucracy in ARC4.

Maynard's framework was an individual model and needed adaptation to this collaborative context. Collective empowerment seemed contingent on a collective sense of proactivity or re-action, on collaboratively agreed goals, shared commitment and multiprofessional team development. In the data there were positive and negative examples of each of these. Volunteering to participate in action research showed one team's proactivity, commitment, aspiration for better integrated care and process of multiprofessional learning (ARC3). The opposite was also true and many teams spoke of working passively in the face of oppressive policies, no agreed goals in integrated teams, and no opportunity to learn or develop (ARC1 and ARC2). These teams were disempowered.

**Figure 3.**
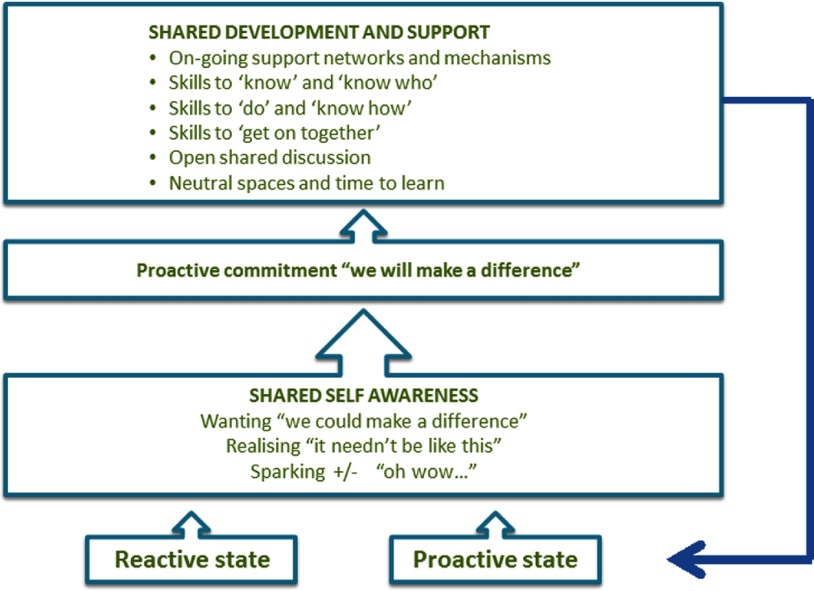
‘Empowerment’ in the collaborative agency for integrated care model.

Constant change in membership, groups, organisations, settings and structure meant that empowerment was not stable. Teams may feel empowered one week, and disempowered the next, returning to the start of the empowerment process. This was reflected in Maynard's original model with ‘recycling’. Collective empowerment is therefore a cycle itself within the collaborative agency model, where the team can return to a proactive or reactive state at any time, or in any setting. This process of empowerment is shown in Figure 3 and draws extensively on Maynard.

Empowerment was critical in each level of the contextual structure, as the empowered teams felt more able to take on structural barriers. Empowerment also related to identity as a team’ collective identity shaped how empowered they felt. Empowerment also determined whether people would take action or not, and so was positioned as the third item in the model, determining agency.

### Collaborative agency

As collaboration is an action the data were filled with references of people taking action and being unable to act. Data showed that the practitioner's agency could be enhanced or constrained by the contextual structures that they operated in, and there were also examples of agency overcoming structures. The following quote encapsulates both of these possibilities: ‘The rule I was given from above when I first attended was ‘go but don't do anything, just show your face’. It was an awful steer, but then this was when … working out the team was so difficult! I wasn't happy as I like to do, so I got involved. Now things have changed in the police and I am more in control, my rule is to only go to meetings if you CAN do something, if not you need to pull out’ (ARC2).

Sociological literature revealed the concept of action to be linked to ‘agency’. The term ‘agency’ refers to the intentional choices and actions of an individual in the world. It is often referred to as human agency or personal agency to discriminate it from ‘agencies’. Agency; ‘implies the ability of individuals or groups to act on their situations, to behave as subjects rather than objects in their own lives, to shape their own circumstances and ultimately achieve change’ [47]. This involves awareness, choice and action. The outcome of these understandings, choices and actions does not need to be positive, or achieve the goal that the person had in mind, in order for them to have agency – it is enough that they thought, chose and acted. The option of not intervening is also a sign of agency. This notion that inactivity is also an ‘act’ is an important if subtle distinction, and research participant's seemed clear that deciding ‘not to’ act was often a positive and difficult choice; ‘Well we had to do nothing, it was the best option, but it didn't feel comfortable as we all like to ‘do’ things!’ (ARC1). Adversity as well as opportunity levered practitioner's agency: ‘Oh if someone says to me that it can't be done, then I have to do it. Like everyone said that I would not get into X academy, so I got on the phone to people I knew and was like ‘hello, it's me, I can do this, wouldn't it be great…’ and I got in there, I'm persistent, coercive, I do what I need to do to get a good outcome’ (ARC4).

Caldwell [48] introduced a range of skills that he considered were important in enacting change agency: consciously choosing, using power, and reflexively initiating, enacting, directing or managing. This introduces the importance of making decisions, of choosing.

Agency comprised three parts: being aware (as in a Frierian sense of critical consciousness [49] or as in Ledwith's description of critical pedagogy [50]), making choices, and taking actions. These three components were all apparent in the data. The participants were aware of the importance of awareness as the following excerpt shows: ‘and they need to be politically astute, it's not about ignoring the structures and the power, it's about being able to use them in the right way, like [name] knowing when it was appropriate to move forwards by going direct to the board’(ARC3). Following this awareness, effective agents then needed to analyse their available choices: ‘Analysis is about pulling out the key issues, potential, strengths, issues and reasons for the conclusions that you have come to’ (ARC3). Taking action seemed to be the most difficult of the aspects of agency, as alluded to in the following data: ‘An appetite for risk is also apparent, If we think about that last task, there was no passion, there was commitment but no action, and no appetite for risk, that's why nothing progressed’ (ARC3). Perhaps because of this, one of the participants concluded: ‘We need more leaders with human agency who can overcome organisational constraints and see that wider picture’ (ARC4). The ability to move beyond the rhetoric of integrated care, into action was important and fostered by responsibility and accountability within teams.

Anthony Giddens [51] structuration and Margaret Archer's [52, 53] double morphogenesis models conceptualised agency and were compared to the data sets. Both these models claimed to overcome the dualism of structure versus agency by presenting a duality. The duality resonated with this research, but both Giddens and Archer's models were individual rather than collective accounts of structure and agency, justifying the new model proposed here. Another issue was that neither of these models used language that was accessible to practitioners, and both existed at an abstract rather than practical level. This reinforced the need for this research to speak at a practice level. Archer's model described ‘personal emergent powers’ that increased an individual's agency. These powers resonated with the data on empowerment discussed previously. Practitioners needed empowerment rather than position to have agency: ‘People don't necessarily need the right position in an organisation, as long as they have the personal power or influence to make things happen’ (ARC4).

Edwards studied interprofessional learning and found that the ‘modernity’ of the modern workplace has increased interconnectedness and personal responsibility, as such;

Arguably strong forms of agency are required to help people such as practitioners who need to collaborate across organisational boundaries, to find moments of stability as they move in and out of different settings without the protection of institutional shelter. [54]

She proposed ‘relational agency’ as a particular type of agency required for working in interprofessional groups, but the concept was presented as an individual attribute rather than a collective capacity. As integrated care requires collaboration, and the data were collective, the model needed to show collaborative, not individual agency. There were multiple examples in the data of individual agency railroading collaborative agency if agendas were not aligned (ARC 1, 2, 3) and so becoming a collective was a key step in collaborative agency, reinforcing the importance of multiprofessional identity collective empowerment and now, collaborative agency.

Dialogue was a key component of collaboration. It worked best when it was equitable with all the members able to speak and be heard. Shared language facilitated understanding in this dialogue, as did time. This action research had afforded participants the time, perspective and tools to develop such a collective awareness. The awareness needed to comprise three elements – there was the need for the team to analyse the context that they were situated in, to identify the possibilities for action and the forces that might enable or constrain them. In other words, teams needed to identify and negotiate the structures identified in Figure 1, negotiating risk. The possibilities for action would be directly influenced by the collective multiprofessional identity that the team has (Figure 2) and their sense of empowerment (Figure 3).

**Figure 4.**
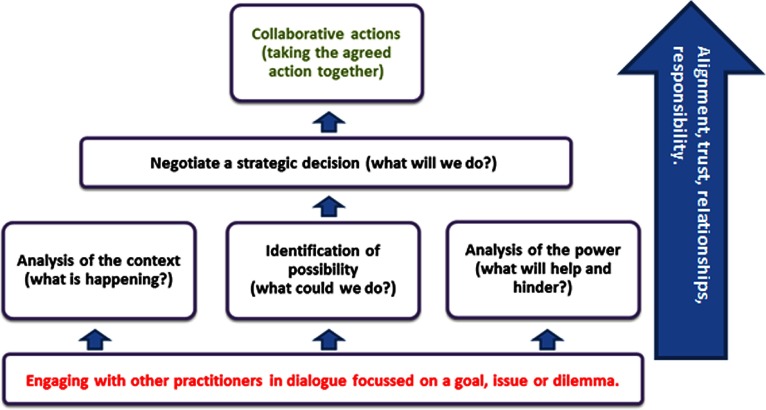
‘Collaborative Agency’ in the collaborative agency for integrated care model.

From the position of awareness, the teams could make joint decisions about what to do. This incorporated leverage of the enabling factors and ways to overcome constraints (ARC4). Strategic decisions to act were easier when they were aligned to values (ARC3), when there was trust in the team (ARC4), and when all the members got on and were able to take responsibility for themselves (all ARC's). Agreeing on a course of action and all carrying out the agreed tasks seemed the most difficult aspect of the work of many of the team's in the study as they often had conflicting priorities from their home organisation (ARC1 and RC3). These elements are shown in Figure 4.

When all the factors were in place (effective dialogue, good awareness, a strong collective multiprofessional identity, a sense of identity, effective decision making and collaborative actions based in trust) collaborative advantage was achieved [55]. The opposite was also true, and collaborative inertia could be seen to occur when practitioners did not engage together, when analysis was flawed or limited by single professional perspectives, when decisions were neither strategic, nor negotiated and shared, or when individuals did not take the agreed actions.

Collaborative agency happened in a context, and was influenced by identity and empowerment. The enactment of agency in turn could lead to changes to the structural context, either reinforcing or changing it. Collaborative agency therefore completed the cycle in the Integrated Care Model.

### The collaborative agency for integrated care model

When put together, structure and agency at multiple levels, agency, and empowerment constitute a dynamic system of collaborative agency. A context pre-exists action. That context comprises the entwined existing structures and existing enactment of agency across four levels. Those are the level of beneficiaries, practice, organisation or profession and overt national policy and covert expectations on ways of being. How we respond to this situation is dependent on our sense of professional self. This is influenced by the success or otherwise of our past actions, and our interactions with other people at the each of the four levels of the context that we act in. This happens at an individual and collective level. Professional identity is always changing in different circumstances and at different times, it is not fixed. We respond to the context and our sense of our professional selves, and can be reactive or proactive to it. The context may ‘spark’ us to make positive gains, or avoid negative outcomes, and we realise that we want to achieve a goal through our work together and proactively commit to that shared goal together. The context, our professional identity and the extent to which we are empowered or not all affect our agency. The collective may engage in work together, tackling a shared goal, where they analyse the context, possible courses of action and sources of power, before negotiating what they do together and taking joint action. A limited professional identity or may prevent us from identifying or adopting a full range of actions. Limited skills may mean that our analysis is weak, or that we cannot enact the actions that we have identified, or that we do not have the skills to work with the people in the other levels of the system. The opposite is also true. Individuals possess agency within the collective, and the difference between individual and group agency may, or may not be a cause of tension. The enactment of agency will lead to some degree of change in the context. The changes in the four structures affect the professional identities of all involved, so structure and agency shift in response to an enactment of agency, and the cycle re-commences.

**Figure 5.**
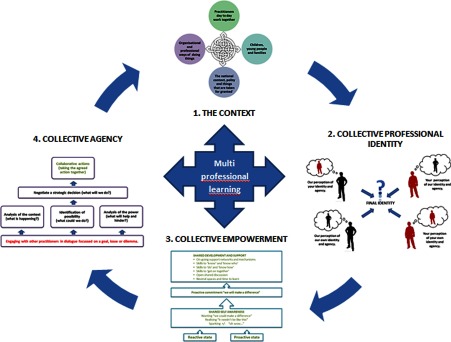
Collaborative agency for integrated care.

The full model is shown in Figure 5.

There are some limitations to the model. The primary limitation is that this is, as yet, an untested model. Feedback would suggest that practitioners view it as useful and practical; ‘its beauty is that it captures a complex social practice and conveys it in a simple and practical way’. However, there is yet no evidence to show its success as a process for collaboration, or in improving integrated care. As stated in the methodology, the other limitation of the model is that it is highly contextualised, it has come from study in one local authority in the UK, and only application and analysis in other contexts will demonstrate whether it has wider use. The model does seem applicable in contexts that are wider than the children's workforce.

Another limitation could be the prevalence of models of integration. Given the number of diagrams and models in England there would hardly seem to be the need for more. However, most of the existing models are for technical tasks that professionals need to carry out, such as the Department for Education model of levels of needs, lovingly called ‘the windscreen model’ [56]. Other models expressed the centrality of the needs of children, such as ‘Getting it Right for Every Child’ [57]. No models were found in the process of literature review that focussed on the needs of the professionals who were bound in the process of delivering integrated services to meet the needs of children, young people and families. This is the niche from which this model emerged, and at which this model is targeted.

As a model of collaboration, it would appear to have relevance to any context where people are collaborating, within an organisation, across organisations or across countries. Indeed, the United Nations conference on child migration and trafficking [58] considered the international possibilities of the model helping countries tackle issues together that cross geographic boundaries, such as child trafficking. The model can be used to help plan for new collaborative endeavours or to evaluate the process of collaboration in established contexts. The model has also been applied in one organisation as a process consultancy tool, used to assess the needs of organisations in the process of commissioning organisational development. The model of collaborative agency is in its infancy however, it represents what practitioners in one local authority believe to be necessary for them to provide effective integrated care, but whether it does so or not is the domain of future research.

## Conclusion

The overarching implication of the model presented is to ensure that collaborative agency is deliberately planned for, fostered and supported. This can be initiated by an individual within the team, but needs to be collectively owned and engaged in. The dividends of investing time and energy into the development of collaborative agency will be reaped as such teams will then be able to move more quickly towards collaborative advantage. There are however no quick fixes, or easy wins, collaboration is a deliberate and demanding task that can perhaps be guided by this model.

This model seems to overcome the assumptions that are commonplace about collaboration, it does not assume that it is simple, cost effective, or the only way to solve problems. Rather the model helps practitioners themselves to manage the complexity and ambiguity inherent. The model present a sociocultural model of collaboration that accounts for history, individuality and the collective, as such, it is more nuanced and sophisticated than protocols offered by policy to date. Further, developing collaborative agency can be seen as encouraging professionals to reclaim their professionalism, their agency, changing the prevalent negative discourses at play in the children's workforce in the UK today.
